# Ensemble learning-based approach for automatic classification of termite mushrooms

**DOI:** 10.3389/fgene.2023.1208695

**Published:** 2023-10-11

**Authors:** Thi Kim Chi Duong, Van Lang Tran, The Bao Nguyen, Thi Thuy Nguyen, Ngoc Trung Kien Ho, Thanh Q. Nguyen

**Affiliations:** ^1^ Department of Information Technology, Lac Hong University, Dong Nai Province, Vietnam; ^2^ Faculty of Engineering and Technology, Thu Dau Mot University, Binh Duong Province, Vietnam; ^3^ HUFLIT Journal of Science, Ho Chi Minh City University of Foreign Languages and Information Technology, Ho Chi Minh City, Vietnam; ^4^ Department of Railway-Metro Engineering, Ho Chi Minh City University of Transport, Ho Chi Minh City, Vietnam

**Keywords:** ITS, molecular biology, DNA barcode, termite mushrooms, termite fungal taxonomy, ensemble learning

## Abstract

Termite mushrooms are edible fungi that provide significant economic, nutritional, and medicinal value. However, identifying these mushroom species based on morphology and traditional knowledge is ineffective due to their short development time and seasonal nature. This study proposes a novel method for classifying termite mushroom species. The method utilizes Gradient Boosting machine learning techniques and sequence encoding on the Internal Transcribed Spacer (ITS) gene dataset to construct a machine learning model for identifying termite mushroom species. The model is trained using ITS sequences obtained from the National Center for Biotechnology Information (NCBI) and the Barcode of Life Data Systems (BOLD). Ensemble learning techniques are applied to classify termite mushroom species. The proposed model achieves good results on the test dataset, with an accuracy of 0.91 and an average AUCROC value of 0.99. To validate the model, eight ITS sequences collected from termite mushroom samples in An Linh commune, Phu Giao district, Binh Duong province, Vietnam were used as the test data. The results show consistent species identification with predictions from the NCBI BLAST software. The results of species identification were consistent with the NCBI BLAST prediction software. This machine-learning model shows promise as an automatic solution for classifying termite mushroom species. It can help researchers better understand the local growth of these termite mushrooms and develop conservation plans for this rare and valuable plant resource.

## 1 Introduction

Termitomyces mushrooms are a type of mushroom that nature has gifted us, known for their high nutritional value and delicious taste (Pegler, 1994). In addition to its high nutritional value, this termite mushroom is also known for its medicinal properties in many countries around the world. Termitomyces mushrooms have antibacterial properties, such as *Termitomyces clypeatus* against *Pseudomonas aeruginosa*, *Termitomyces eurhizus* against *Proteus vulgaris* and *Scherichia coli*, and *Termitomyces microcarpus* against *Bacillus cereus* and *Proteus vulgaris* (Giri, 2012). *Termitomyces clypeatus* also supports the treatment of chickenpox (Dutta and Acharya, 2014). The valuable compounds of these rare and valuable mushroom species are obtained through biomass cultivation (Lu et al., 2008) cultivated *Termitomyces albuminosus* to test its efficacy in pain reduction and anti-inflammation while *Termitomyces striatus* was used for other extracted compounds. *Termitomyces heimii* and *Termitomyces microcarpus* are used in the treatment of fever, colds, and fungal infections and in promoting cancer therapy (Venkatachalapathi and Paulsamy, 2016). There are about 30 species of Termitomyces mushrooms worldwide, and 10 species in Vietnam, with *Termitomyces clypeatus* and *Termitomyces microcarpus* being common in Binh Duong. Although very effective economically, the natural yield of these mushrooms is declining significantly, and they have not yet been cultivated sustainably, as they only grow seasonally.

Correctly identifying the name of a termite fungus species is an important task in biological research. Experts use traditional methods to classify and identify termite fungi based on their morphology. The overall structure of a termite fungus includes a cap, flesh, membrane, and stem, which may have rings and boxes (Mossebo et al., 2009). However, fungal structures vary from species to species, especially when mutations occur. Moreover, identifying samples lacking morphological characteristics can be difficult (Roe et al., 2010). A method for identifying new species of organisms that are often used to identify edible and medicinal mushrooms is based on molecular techniques. In this approach, molecular techniques such as DNA barcoding have been successfully used in recent years to identify species (Hebert et al., 2003; Somervuo et al., 2016). These molecular methods are based on analyzing genetic markers and have proven to be highly effective in identifying species, especially when combined with traditional morphological methods. Overall, incorporating molecular techniques into the identification process of termite fungi can provide more accurate and efficient identification, especially in cases where traditional morphological methods fall short.

**FIGURE 1:**
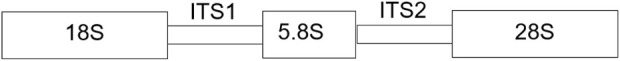
The ITS sequences region (White et al., 1990).

One commonly utilized gene group in molecular identification is the group that encodes rRNA. This group is highly effective for finding similarities and differences when comparing different organisms due to the relatively conserved nature of most rRNA molecules (De Peer et al., 1996). For fungi, the rDNA ITS (Internal Transcribed Spacer) region, which includes two sequences, ITS1 and ITS2, flanking the 5.8S sequence, is widely accepted as the molecular region for species identification by most mycologists (Kõljalg et al., 2013), as shown in Figure 1. The ITS region is also used for predicting fungal species using machine-learning. This approach involves using the ITS sequence data to train a machine-learning model, which can then be used to accurately classify and identify different fungal species automatically. By combining molecular techniques such as machine-learning with traditional morphological identification methods, researchers can achieve more accurate and efficient identification of fungal species, aiding in both research and conservation efforts.

The ITS sequence data for fungi can be accessed from two major datasets, BOLD (Barcode of Life Data) and the National Center for Biotechnology Information (NCBI). Both contain a vast collection of ITS sequences for all fungal species. Machine learning-based classification of fungal species using ITS sequences has been proposed by several researchers, including (Schloss et al., 2009; Schoch et al., 2012; Delgado-Serrano et al., 2016; Deshpande et al., 2016; Edgar, 2016; Meher et al., 2019; Das et al., 2023). A comprehensive list of the techniques and data used in fungal classification studies is provided in Table 1.

**TABLE 1:** Relevant works that used machine-learning based on ITS dataset.

References	Tool	No. of sequence per category	The source of barcode sequences of fungal species	Feature technical and ML algorithm	Accuracy of the best model
Schloss et al. (2009)	MOTHUR	-	The SILVA Database Project, Bremen, March 2009	K-mer (k = 5), The k-nearest neighbor (kNN) algorithm, and PGMA (unweighted-pair group method using average linkages) algorithms	0.86
Delgado-Serrano et al. (2016)	Mycofar	-	NCBI GeneBank	K-mer (k = 5), Naïve Bayes classifier	0.87
Deshpande et al. (2016)	RDP	10	The Warcup dataset (18878 sequences belonging to 8551 species)	K-mer (k = 8) Bayesian regression.	0.87
Edgar (2016)	SINTAX	14	RDP Warcup ITS (18878 sequences belonging to 8551 species)	K-mer (k = 8)Naive Bayesian Classifier	0.87
Meher et al. (2019)	funbarRF	10	BOLD systems	K-mer (k = 4) Random Forest.	0.89
Das et al. (2023)	CNN_FunBar	20	UNITE + INSDC (4504529 sequences belonging to 44167 species)	K-mer (k = 6), CNN	0.86

The mentioned studies have successfully utilized supervised machine-learning techniques such as Naive Bayes classification, kNN, and Bayesian regression models for classifying fungal species. However, only (Delgado-Serrano et al., 2016) identified the fungal species at the genus level, while other studies only determined the species names. As ITS sequence data from the NCBI GeneBank were used, this data is not sufficient for identifying the labels of termite fungi found in these GenBank. For example, the ITS sequence of the termite fungus genus *Termitomyces euripus* in the NCBI GeneBank has only one sequence, while there are six labels for this fungal genus in BOLD. Additionally, the lengths of ITS sequences vary widely, ranging from 200 bases to 2000 bases, and the number of sequences between fungal genera varies greatly, from one to 500 sequences. Due to these limitations with ITS data for termite fungi, classical machine-learning algorithms struggle to accurately classify the labels of termite fungi. Our study focuses on identifying the labels of termite fungal genera using ITS sequence data collected from both the NCBI GeneBank and BOLD GenBank. The K-mer technique and natural language processing (NLP) were combined to extract features, and modern classification methods such as XGBoost (Extreme Gradient Boosting), Random Forest, and CatBoost are experimented with to build an automatic termite fungal species classifier. The proposed research is structured as follows: the method presents the concepts related to ITS sequence data, feature extraction techniques, the overall proposed model, experimental results, and finally, the study’s conclusion.

## 2 Methods

### 2.1 ITS sequence data

Termite fungi are valuable but endangered, and urgent research and conservation efforts are needed. However, data on ITS sequences for termite fungi in GenBank are incomplete, making it crucial to synthesize data from different sources. In this article, ITS sequence data from two GenBank, BOLD and NCBI, was compiled by us. Specifically, 101 ITS sequences were obtained from BOLD, with the number of sequences for each genus ranging from 1 to 12. At NCBI, 1740 ITS sequences were obtained, with the number of sequences for each species ranging from 1 to 799. After synthesizing the ITS sequence data from these two GenBank and removing termite fungal species with fewer than 7 sequences, 1704 sequences belonging to 17 termite fungal species were obtained. The labels of each termite fungal species are presented in detail in Table 2. This data can be used for further research and conservation efforts for these valuable and endangered fungi.

**TABLE 2:** Termitomyces species used for the training dataset.

No	Termitomyces species label	No. of sequences	Lable
1	*Uncultured Termitomyces*	799	16
2	*Termitomyces* sp.	483	10
3	*Termitomyces intermedius*	94	8
4	*Termitomyces symbiont*	60	14
5	*Termitomyces microcarpus*	34	9
6	*Termitomyces clypeatus*	33	3
7	*Termitomyces cylindricus*	30	4
8	*Termitomyces striatus*	29	13
9	*Termitomyces DKA-2007*	24	0
10	*Termitomyces heimii*	24	7
11	*Termitomyces bulborhizus*	17	2
12	*Termitomyces fuliginosus*	16	6
13	*Termitomyces eurrhizus*	15	5
14	*Termitomyces albuminosus*	14	1
15	*Termitomyces* sp. symbiont of *Macrotermes bellicosus*	12	11
16	*Termitomyces* sp. symbiont of *Macrotermes subhyalinus*	10	12
17	Uncultured Ascomycota	10	15

The ITS region of termite mushrooms collected from Binh Duong province, Vietnam, was sequenced, and the resulting sequences have a length ranging from 669 to 1050 base pairs. These termite mushroom samples have a morphology similar to that of *Termitomyces clypeatus, Termitomyces microcarpus* and *Termitomyces striatus.* The sequence data for these eight termite mushroom samples have been published and stored in the NCBI GeneBank. For more detailed information about these termite mushroom samples, please refer to Table 3.

**TABLE 3:** Information of Termitomyces species in Binh Duong Province, Viet Nam.

ID_sequences	Binh Duong termitomyces species in NCBI	Website	Length of sequences
KU569480	*Termitomyces clypeatus*	https://www.ncbi.nlm.nih.gov/search/all/?term=KU569480	980
MF163136-BD5	*Termitomyces clypeatus*	https://www.ncbi.nlm.nih.gov/search/all/?term=MF163136	720
MF163152.1	*Termitomyces clypeatus*	https://www.ncbi.nlm.nih.gov/search/all/?term=MF163152	938
MF163445-BD3	*Termitomyces* sp.	https://www.ncbi.nlm.nih.gov/search/all/?term=MF163445	669
MF163446-BD6	*Termitomyces* sp.	https://www.ncbi.nlm.nih.gov/search/all/?term=MF163446	1020
MT672480.1	*Termitomyces microcarpus*	https://www.ncbi.nlm.nih.gov/search/all/?term=MT672480.1	721
MT730584.1	*Termitomyces clypeatus*	https://www.ncbi.nlm.nih.gov/search/all/?term=MT730584	608
MF163149-BD4	*Termitomyces* sp.	https://www.ncbi.nlm.nih.gov/search/all/?term=MF163149	812

### 2.2 Feature generation

**FIGURE 2:**
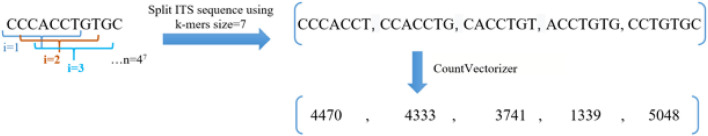
Illustrate the use of the K-mer method to encode ITS sequences into numeric vectors, in the example the size of K-mer was 7.

The extraction of features from biological sequences is a crucial step in computational biology. Biological sequences are typically composed of a string of letters, which must be converted into numerical vectors before they can be utilized in machine-learning algorithms (Kamath et al., 2014). The K-mer feature technique has been employed to represent information for ITS sequences to classify species based on barcodes, as demonstrated by previous studies (Schloss et al., 2009; Deshpande et al., 2016). In 2016, Delgado-Serrano utilized K-mer encodings to transform ITS sequences into numerical vectors. The accuracy of the prediction model was affected by the size of the K-mer utilized (Delgado-Serrano et al., 2016). In our proposed approach, a combination of K-mer and CountVectorizer techniques was employed to encode ITS sequences into numerical vectors. An illustration of the methodology utilized to digitize sequence information is presented in Figure 2.

In Figure 2, The process of digitizing ITS sequences has been illustrated. This process is similar to that of using Natural Language Processing (NLP) tools from Sklearn to convert our K-mer words into numerical vectors. These vectors, which represent the count of each K-mer in the vocabulary, have the same length as unigrams.

### 2.3 Ensemble learning

Supervised machine-learning techniques are widely used in computational biology to solve various problems. Several traditional machine-learning algorithms such as k-nearest neighbors, Naïve Bayes, and decision trees have been successful in identifying mushroom species based on barcode data (Schloss et al., 2009; Delgado-Serrano et al., 2016; Deshpande et al., 2016). However, these models have relatively low accuracy. In our research, two solutions were tested: i) The first set utilized well-known classification methods like Naïve Bayes and Random forest to predict the names of termite mushroom species; ii) In the second, automated models for predicting termite mushroom species with higher accuracy were built by us using Ensemble learning algorithms such as XGBoost and CatBoost.

### 2.4 Gradient-boosted decision trees (GBDTs)

Gradient Boosting Decision Trees (GBDT) (Friedman, 2001) is a method that uses decision tree ensembles to predict target values. A GBDT is constructed by splitting observations based on the attribute values of the input data. The model can find the best way to divide data and determine the most time-consuming part of the partitioning process. To build a GBDT model with T trees from a dataset consisting of n samples, the prediction process according to the GBDT method is as follows:

$$\begin{array}{l} {\hat{y}}_{i}^{\left(0\right)}=0\\ {\hat{y}}_{i}^{\left(1\right)}=f_{1}\left(x_{i}\right)={\hat{y}}_{i}^{\left(0\right)}+f_{1}\left(x_{i}\right)\\ {\hat{y}}_{i}^{\left(2\right)}=f_{1}\left(x_{i}\right)+f_{2}\left(x_{i}\right)={\hat{y}}_{i}^{\left(1\right)}+f_{2}\left(x_{i}\right)\\ ..\\ {\hat{y}}_{i}^{\left(K\right)}=\sum_{k=1}^{K}f_{k}\left(x_{i}\right)={\hat{y}}_{i}^{\left(K-1\right)}+f_{K}\left(x_{i}\right) \end{array}$$ (1)

where ${\hat{y}}_{i}^{\left(K\right)}$ is the predicted value of the *i* ^*th*^ sample at the *k* ^*th*^ iteration

The cost function of GBDT has two parts: a training error and regularization, as follows:

$$\text{Cost}=\sum_{i=1}^{n}l\left(y_{i},{\hat{y}}_{i}\right)+\sum_{k=1}^{K}\Omega \left(f_{k}\right)$$ (2)

where $\Omega \left(f_{k}\right)=\gamma T+\frac{1}{2}\lambda {\left\|w\right\|}^{2},\forall k=\bar{1,K}$ . T is the number of leaf nodes, w is the score for a leaf node, γ is the leaf penalty coefficient, and ensures that leaf nodes’ scores are not too large.

#### 2.4.1 CatBoost

CatBoost is an algorithm used to boost gradients on decision trees. It is used to process datasets with a large number of input features of the categorical data type (Prokhorenkova et al., 2018). In the field of computational biology, CatBoost has been applied for various purposes such as identifying bacterial genes at the 16S rRNA level (Meharunnisa and Sornam, 2022) or building a feature extraction package for DNA, RNA, and protein sequences (Robson, 2022). Our proposals have used the CatBoost algorithm to build a model for termite mushroom species classification.

#### 2.4.2 XGBoost

XGBoost is a powerful machine-learning algorithm that builds upon the initial gradient-boosting machine (Friedman, 2001; Chen et all., 2015), is an upgraded version of gradient boosting that boasts many superior improvements (Ren et al., 2017; Jiang et al., 2019); (Zhong et al., 2018). These improvements, achieved through parallel computation on different datasets, have significantly increased processing speed, making XGBoost up to 10 times faster than GBM. XGBoost has been successfully applied in many fields, including computational biology.

### 2.5 Building the best classifier base on ensemble learning

CatBoost is a viable option for gene sequence data analysis, as indicated by recent research (Robson, 2022). In our experiments with termite mushroom data, it was observed that CatBoost performed comparably to XGBoost in terms of prediction accuracy. However, a relatively longer training time is required by CatBoost than that of XGBoost to achieve a similar level of performance. Therefore, XGBoost was chosen as the primary algorithm for our prediction model.

The XGBoost model’s performance depends on several key parameters such as *'max_depth*', '*gamma*', *'n_estimators'*, and '*learning_rate'*. These parameters are known as hyperparameters and can be adjusted manually during training or automatically. The proposed enhanced model uses the Bayesian Optimization technique (Klein et al., 2017) specifically Random search, to tune the hyperparameters. Bayesian Optimization was applied to tune the four main parameters of the XGBoost classifier: 'max_depth', 'gamma', 'n_estimators', and 'learning_rate'.

To improve the predictive performance of the model, cross-validation with k = 5 was performed to select the best classification model, in addition to using Bayesian Optimization to tune hyperparameters. A new dataset, which consisted of n data samples and m features, was obtained from the results of phase 1. An optimization parameter was then used as input for Algorithm 1 to build an optimal classification model.

Algorithm 1. Building the best XGBoost classifier

**Input:** $D_{Ter}=\left\{\left(x_{i},y_{i}\right)\in R^{m}\times \left\{0,1\right\},\forall i=\bar{1,n}\right\}$ ; hyperparameter is *Ɵ* = {'max_depth': int(max_depth), 'gamma': Gama, 'n_estimators': int(n_estimators), 'learning_rate':learning_rate }
**Output:** Best_Model
Begin
** **1: **Initialize**: FeatureImportances={}
** **2: Model ← **XGBoostClassifier (** *Ɵ* **)**
** **3: KFold← StratifiedKFold (n_splits=5, shuffle = True, random_state=2020)
** **4: **For** i=1 **each** KFold
**  **• Divide the $D_{Ter}$ dataset into $D_{Train}$ and $D_{Test}$

**  **• Train the model based on early-ending hyperparameters
** **5: **Calculate** the roc_auc_score, accuracy_score, precision_score, recall_score, and f1_score over iterations
** **6: **Select** the best model based on Step 4
** **7: **Visualize** the mean value from Step 4
** **8: **Return** the Best_Model from Step 4
End

### 2.6 Building a model for predicting the termite fungus species name

**FIGURE 3:**
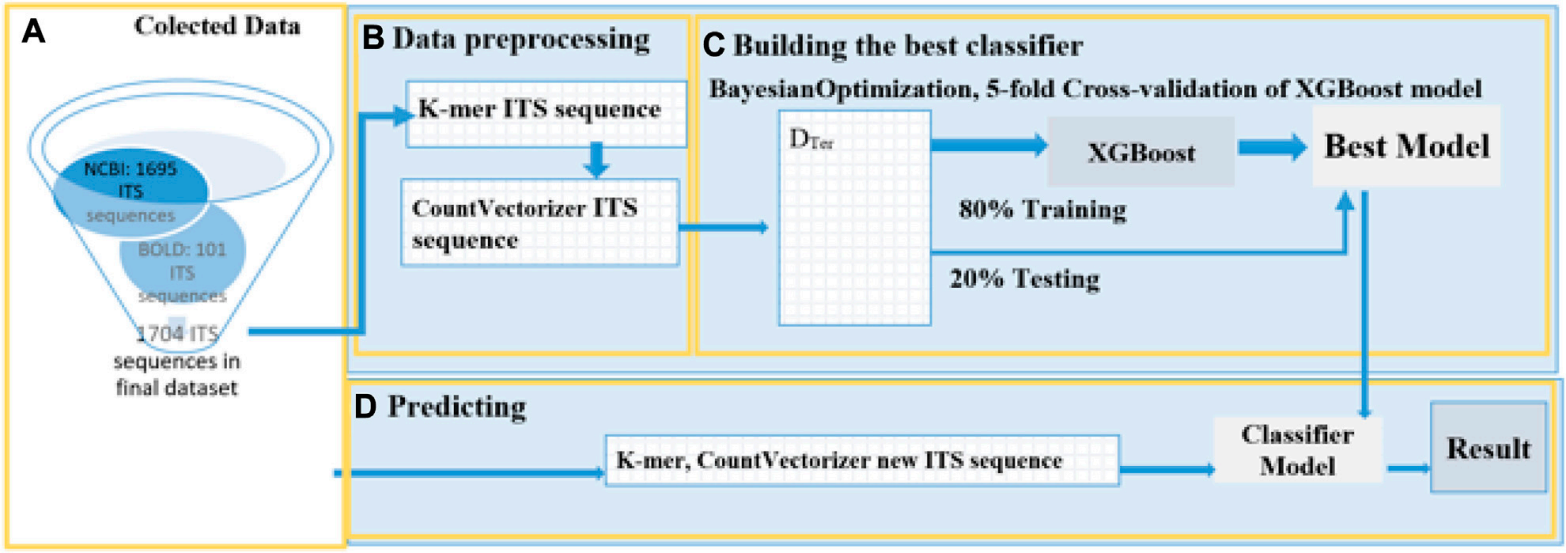
Detailed model of the proposed method. **(A)** Collected Data: The study collected a total of 1796 ITS sequences of mushroom fungus from GenBank NCBI and BOLD. After filtering out termite fungus species sequences with less than 10 sequences, the final count of ITS sequences was 1704. **(B)** Data Preprocessing: The ITS sequences were split into smaller sequences, following the rules described in Figure 2, using K-mer with a size of 7. The longest ITS sequence was 2470 bases, corresponding to a vector length of 14425 when encoded. **(C)**. Training: The training process used an 80:20 split ratio and employed hyperparameter optimization for the training model. The model was optimized using the k-fold Cross-Validation technique with k = 5, and BayesianOptimization was performed to fine-tune the following parameters: *'max_depth'*: (5,10), *'gamma'*: (0,1), *'learning_rate'*:(0,1*), 'n_estimators'*:(100,400). The model with the highest accuracy was selected for the classification. **(D)** Prediction: Mushroom samples collected in Binh Duong Province, Vietnam, and downloaded from NCBI were used as the test set. These samples were subjected to K-mer with a size of 7 and then CountVectorizer was applied. Finally, the best model from stage c was applied to predict the species of new termite fungi.

Our study has developed an automated process consisting of four stages to predict the species name of a new termite fungus. The first stage involves collecting termite fungus data from ITS gene sequence repositories. In the second stage, sequence features are extracted and encoded. The third stage involves building a classifier by constructing and tuning parameters to find the optimal classifier. Finally, in the fourth stage, the classifier is used to predict new termite fungus samples. Figure 3 provides a detailed description of this process.

### 2.7 Performance metrics

In our study, the terms “*true*” and “*false*” predictions can arise from the model’s misclassification or failure to predict accurately, such as false negatives or false positives, or other concepts applied to the prediction targets. Specifically, the phrase “*predicting the species of Termitomyces*” is referred to as a true positive (TP), while the phrase “*correctly excluding the species of Termitomyces*” is referred to as a true negative (TN). On the other hand, the phrase “*predicting the species of Termitomyces incorrectly*” is designated as a false positive (FP), and a “*missed or misclassified prediction*” is considered a false negative (FN). These conditions are utilized as stopping points during initial data training. To evaluate the performance of our proposed model, various methods were applied to assess its machine-learning abilities on DNA sequence data (Gupta, P., et al., 2021). These methods include the following:

❖ Accuracy: The proportion of correctly predicted cases is known as accuracy, and it can be calculated using the following formula:

$$Accuracy=\frac{TP+TN}{TP+TN+FP+FN}$$

❖ Sensitivity: Recall (pr) was the hit rate (hit rate), and the true positive rate (TPR) was the ratio of correct positive classifications to the total number of positive and recall cases and it can be calculated using the following formula:

$$TPR=Sensitivity=\frac{TN}{TN+FP}$$

❖ Specificity: True negative (TN) (or specificity in clinical medicine) was the correct exclusion rate out of the total number of negative cases, it can be calculated using the following formula:

$$Specificity=\frac{TP}{TP+FP}$$

❖ False Positive Rate/Fallout (FPR) was an expression of the rate of mislabeling of negative to positive samples across all negative samples, it was calculated by the following formula:

$$FPR=1-\text{specificity}=1-\frac{TP}{TP+FP}$$

❖ Precision: Since the dataset had a larger sample, this led to an imbalanced input dataset for the prediction model. Therefore, we used precision to determine the ratio of actually positive cases to the total number of cases labeled “positive” by the model. Precision is a term that refers to the “deterministic” or accurate positive classification of a model:

$$Precision=\frac{TP}{TP+FP}$$

❖ F1 score: This was defined as the harmonic mean between precision and recall:

$$F1=2\;x\;\frac{Precision\;x\;Recall}{Precision+Recall}$$

❖ Receiver operating characteristics (ROCs) were used to calculate the model’s classification performance in the condition of unbalanced data set classes. A ROC curve was produced for each pair (TPR, FPR) for different thresholds, with each point on the curve representing one pair (TPR, FPR) for one threshold. This curve shows us the relationship between the True Positive Rate (TPR) and the False Positive Rate (FPR). The ROC Curve and the ROC AUC score are important tools for evaluating binary classification models. To evaluate multi-class classifiers, the OvR (One vs. Rest) technique was used, which compares each class with all other classes simultaneously. In this case, one class was chosen to be the “positive” class, while all other classes (the remaining part) were considered “negative” classes. In the experiment, the last label class 16 was selected as the “positive” class and the remaining classes were considered “negative”. In this way, the multi-class classification output was reduced into binary classification, allowing the utilization of all known binary classification metrics to evaluate the classification model.

## 3 Results and discussion

### 3.1 Result of each stage in the proposed process

In the experimental process, Python 3.9 and the libraries Scikit-learn, Biopython, XGBoost, CatBoost, and Bayesian optimization were employed to construct a mushroom classification model following the proposed process depicted in Figure 3. The results of each stage a, b, c, and d are attached.

❖ During stage a: Data was collected through the following steps: (a.1) retrieving data from the NCBI and BOLD GenBanks, which yielded 1740 sequences of 28 mushroom species; (a.2) selecting 17 species that had at least 10 sequences per species.
❖ During stage b: Data preprocessing was performed in two steps: The ITS sequence strings were separated by applying K-mer with a length of *k* = 7, and then the ITS sequences were converted into numerical data by vectorizing them, and the data labels were also converted into numerical values The section provides details on the number of classes and corresponding data.
❖ During stage c: The best prediction model was built, consisting of (c.1) a classification model and (c.2) an optimized set of hyperparameters.
❖ Finally, during stage d: The performance of the proposed model was displayed in step (d.1), while the predictions of eight ITS sequences collected in Thu Dau Mot, Binh Duong province were shown in step (d.2).

### 3.2 Select the appropriate K-mer sizes for the classifiers

**FIGURE 4:**
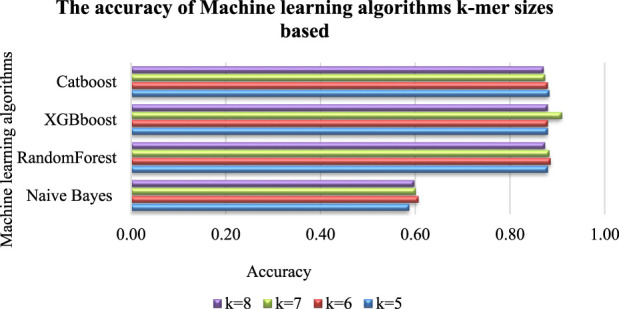
Accuracy of machine-learning algorithms according to the K-mer sizes. It was found that different predictive results (accuracy) were produced by each algorithm for each K-mer size such as: Catboost produced results ranging from 0.87–0.88, XGBboost had results from 0.88–0.91, and RandomForest yielded results from 0.87–0.89. However, the Naive Bayes (MultinomialNB) model had the lowest accuracy, ranging from 0.59–0.61. Figure 5 presents detailed information on the impact of K-mer size on the ROC Curve (AUCROC) achieved by each algorithm. Notably, the XGBoost algorithm achieved the highest classification AUCROC when the K-mer size was set to 7, which was also used to build the automated model for predicting mushroom species names.

**FIGURE 5:**
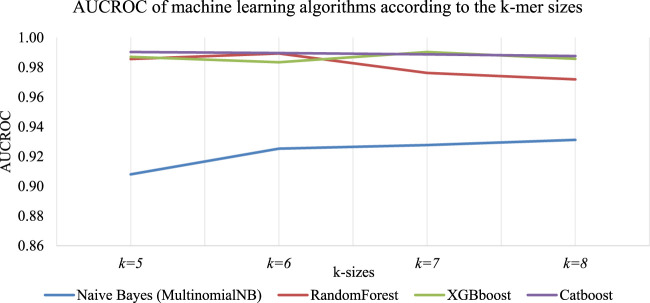
Accuracy of machine-learning algorithms according to the K-mer sizes.

The accuracy of predictive models based on sequence data is significantly impacted by the size of K-mers (Delgado-Serrano et al., 2016). To explore this impact, a study was conducted using different K-mer lengths, which resulted in varying classification accuracies. The sequence in Figure 3 was used to build a classifier, with machine-learning algorithms such as Naive Bayes (MultinomialNB), RandomForest, XGBboost, and Catboost. The classifier’s results for each K-mer size are presented in Figure 4. We found that each algorithm produced different predictive results (accuracy) for each K-mer size. Specifically, Catboost produced results ranging from 0.87–0.88, XGBboost had results from 0.88–0.91, and RandomForest yielded results from 0.87–0.89. However, the Naive Bayes (MultinomialNB) model had the lowest accuracy, ranging from 0.59–0.61. The classifier’s results for each K-mer size are presented in Figure 5.

**FIGURE 6:**
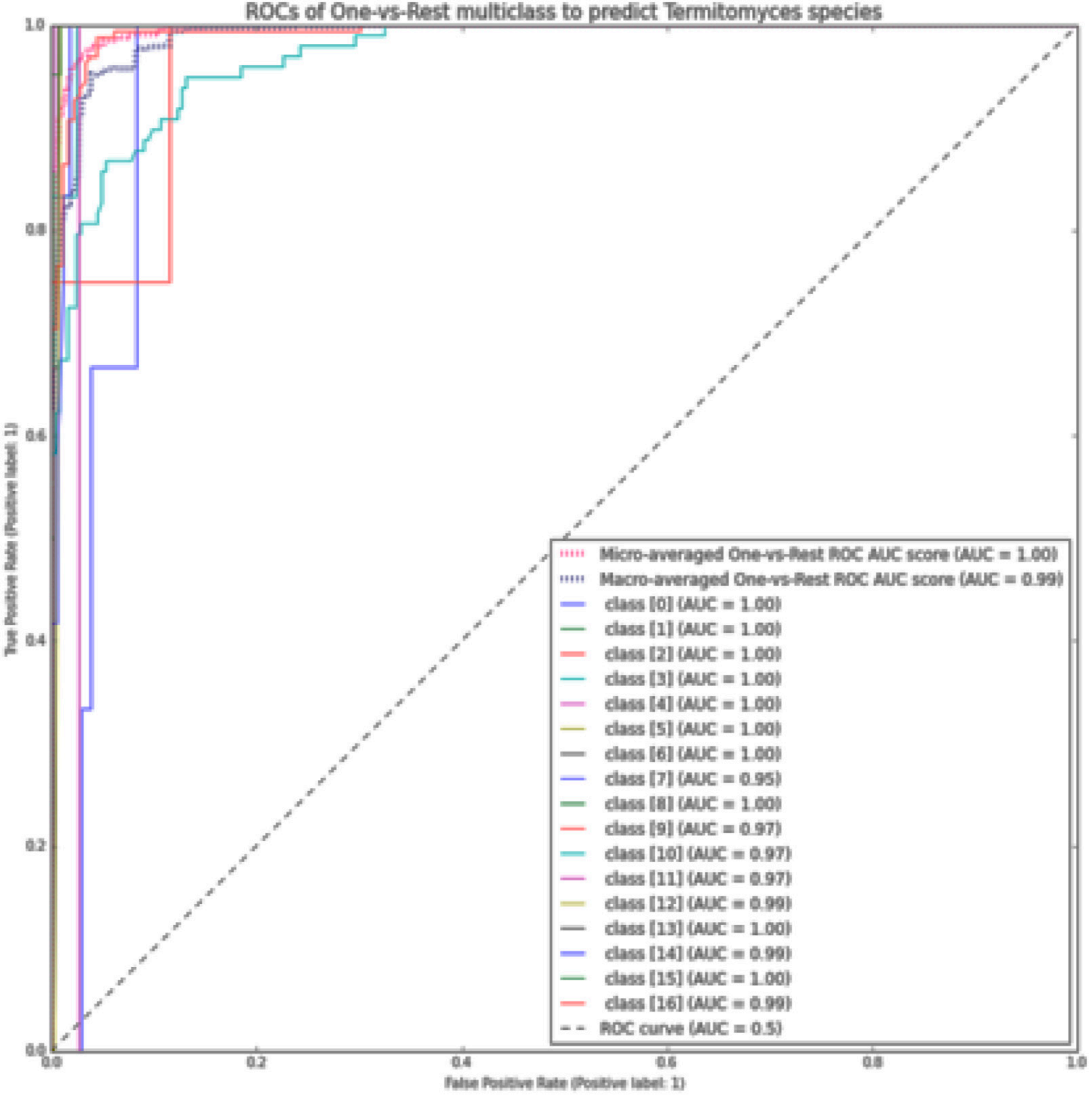
The ROC curve using the OvR macro-average for each class in the XGBoost method by size *K-mer* = 7.

Figure 6 presents detailed information on the impact of K-mer size on the highest accuracy achieved by each algorithm. Notably, the XGBoost algorithm achieved the highest classification accuracy when the K-mer size was set to 7, which was also used to build the automated model for predicting mushroom species names.

### 3.3 Performance analysis in other machine-learning algorithms

Apart from using accuracy as a measure of the classification model’s performance, other metrics such as precision, recall, F1 score, or AUCROC are also utilized to evaluate the classifiers’ performance. A summary of the performance of the surveyed machine-learning algorithms is presented in Table 4.

**TABLE 4:** Synthesize the performance of machine-learning algorithms.

Method	AUROC	Accuracy	Precision	Recall	F1 score
Naive Bayes	0.93	0.60	0.84	0.60	0.62
RandomForest	0.98	0.88	0.88	0.88	0.88
XGBboost	0.99	0.91	0.90	0.91	0.90
Catboost	0.99	0.87	0.87	0.87	0.87

Furthermore, the AUCROC for each class was calculated using the ROC curve method with the OvR macro-average for the multi-class model utilized (Pedregosa et al., 2011). In this study, the last class (class 16) was designated as the positive class, while all other classes were considered negative classes. The visual representations of each class’s results are presented in Figure 6.

### 3.4 Comparative analysis for prediction of fungal species

Previous models for predicting fungal species accuracy have been evaluated using the *K-mer* method and machine-learning techniques such as *k-Nearest* Neighbor, Naïve Bayes, and Random Forest, with results presented in Table 5. Our proposed approach demonstrates superior performance when utilizing a *K-mer* size of 7 with the XGBoost classification algorithm. Table 5 presents a comparison of various classifiers' performance for predicting fungal species using ITS sequences.

**TABLE 5:** Performance comparison of fungal classifiers using ITS sequencing.

Ref	Method	Accuracy
Schloss et al. (2009)	K-mer (k = 5), The k-nearest neighbor (kNN) algorithm, and PGMA algorithms	0.86
Delgado-Serrano et al. (2016)	K-mer (k = 5), Naïve Bayes classifier model	0.87
Deshpande et al. (2016)	K-mer (k = 8) Bayesian regression.	0.87
Meher et al. (2019)	K-mer (k = 4), Random Forest.	0.89
Our proposal	K-mer (k = 7), XGBoost	0.91

### 3.5 Compare the prediction results of the proposed model with the results of BLAST

The ITS sequences of termite fungi collected from Binh Duong province, Vietnam, were published on NCBI and are detailed in Table 3. Our proposed classification model predicted species identification with comparable results to those obtained from NCBI. For instance, sequences MF163150-BD1, MF163151-BD2, and MF163147-BD7 were identified as the same species as those on NCBI. Moreover, the species identification of MF163149-BD4 was consistent with the identification on NCBI. However, for MF163445-BD3, MF163446-BD6, and MF163149-BD4, the identification was previously unknown or unclear. Our proposed classification model successfully identified MF163445-BD3 and MF163446-BD6 as *Termitomyces striatus*, consistent with the type strain of the collected fungi. The results for MF163149-BD4 were also consistent with the species identification on NCBI. Table 6 presents the details of the species identification results.

**TABLE 6:** Result in comparison of the species identification of ITS sequences of termite fungi collected in Binh Duong province, Vietnam, with the identification on NCBI.

ID_sequences	Binh Duong termitomyces species in NCBI	Binh Duong termitomyces species in our proposal
KU569480	*Termitomyces clypeatus*	*Termitomyces clypeatus*
MF163136-BD5	*Termitomyces clypeatus*	*Termitomyces clypeatus*
MF163152.1	*Termitomyces clypeatus*	*Termitomyces clypeatus*
MF163445-BD3	*Termitomyces* sp.	*Termitomyces striatus*
MF163446-BD6	*Termitomyces* sp.	*Termitomyces striatus*
MT672480.1	*Termitomyces microcarpus*	*Termitomyces microcarpus*
MT730584.1	*Termitomyces clypeatus*	*Termitomyces clypeatus*
MF163149-BD4	*Termitomyces* sp.	*Termitomyces* sp.

Accurately identifying new species is crucial for studying biodiversity and formulating conservation policies for endangered species (Van Velzen et al., 2012). Traditional methods of species identification based on physical characteristics can be difficult, prompting the use of DNA barcoding as an alternative approach (Hibbett et al., 2011). In this study, a novel computational method is proposed that utilizes K-mer techniques and NLP vectorization to convert DNA barcode sequence data into digital features. The XGBoost algorithm is then employed to build a model capable of predicting termite mushroom species using the ITS sequence as a DNA barcode.

The performance of the developed model was evaluated on 1704 sequences of 17 mushroom species obtained from two ITS GenBanks. The evaluation was conducted using standard classification metrics such as accuracy, precision, recall, F1-score, and AUCROC.

Our proposed model was assessed by comparing its predictions with the species identification results on NCBI, demonstrating complete consistency with the identified species of the ITS sequences of mushrooms, as well as predicting the species names of two sequences that had not previously been identified. An example of this is the *Termitomyces striatus* mushroom specimen found in Binh Duong province, Vietnam, which was correctly identified by our proposed model. Furthermore, when compared to four other research groups' machine-learning models for predicting termite mushroom species names, our proposed model achieved an accuracy of 0.91 and an average AUCROC score of 0.99, demonstrating its efficacy in species identification. These results suggest that our proposed model is a valuable tool for identifying termite fungi species in Binh Duong province, Vietnam, and could be applied to other mushroom species as well.

## 4 Conclusion

This study presents a computational model to predict termite fungus species based on DNA barcodes. The paper also introduces a new method for creating features based on K-mer techniques, NLP vectorization to digitize sequence data, and an optimized classifier. The results showed that the model was evaluated based on the standard classification systems’ measures, including accuracy, precision, recall, *f1*-score, and AUCROC. The model was evaluated on 17 termite mushroom species and achieved high accuracy when compared with species identification results on NCBI. These results suggest that the proposed model can be an effective tool for identifying termite mushroom species based on DNA barcodes. Furthermore, the proposed method can also be used to predict other species.

## Data Availability

The original contributions presented in the study are included in the article/supplementary material, further inquiries can be directed to the corresponding author.

## References

[B1] Chen T. He T. Benesty M. Khotilovich V. Tang Y. Cho H. (2015). Xgboost: extreme gradient boosting. R. package version 0.4-2 1 (4), 1–4.

[B2] Das R. Rai A. Mishra D. C. (2023). CNN_FunBar: advanced learning technique for fungi ITS region classification. Genes. 14 (3), 634. 10.3390/genes14030634 36980906 PMC10048311

[B3] Delgado-Serrano L. Restrepo S. Bustos J. R. Zambrano M. M. Anzola J. M. (2016). Mycofier: A new machine learning-based classifier for fungal ITS sequences. BMC Res. Notes 9 (1), 402–408. 10.1186/s13104-016-2203-3 27516337 PMC4982325

[B4] Deshpande V. Wang Q. Greenfield P. Charleston M. Porras-Alfaro A. Kuske C. R. (2016). Fungal identification using a Bayesian classifier and the Warcup training set of internal transcribed spacer sequences. Mycologia 108 (1), 1–5. 10.3852/14-293 26553774

[B5] Dutta A. K. Acharya K. (2014). Traditional and ethno-medicinal knowledge of mushrooms in West Bengal, India. Asian J. Pharm. Clin. Res. 7 (4), 36–41.

[B6] Edgar R. C. (2016). Sintax: A simple non-bayesian taxonomy classifier for 16S and ITS sequences. biorxiv.074161

[B8] Friedman J. H. (2001). Greedy function approximation: A gradient boosting machine. Ann. statistics 29, 1189–1232. 10.1214/aos/1013203451

[B9] Giri S. B. (2012). Antimicrobial activities of basidiocarps of wild edible mushrooms of West Bengal, India. Int. J. PharmTech Res. 4 (4), 1554–1560.

[B10] Gupta P. Melkani G. Maggu S. Rathee A. (2021). Genome sequencing and classifier. Int. J. Adv. Eng. Manag. 4 (4), 1554–1560. 10.35629/5252-030617591767

[B11] Hebert P. D. Cywinska A. Ball S. L. DeWaard J. R. (2003). Biological identifications through DNA barcodes. Proc. R. Soc. Lond. Ser. B Biol. Sci. 270 (1512), 313–321. 10.1098/rspb.2002.2218 PMC1691236 12614582

[B12] Hibbett D. S. Ohman A. Glotzer D. Nuhn M. Kirk P. Nilsson R. H. (2011). Progress in molecular and morphological taxon discovery in Fungi and options for formal classification of environmental sequences. Fungal Biol. Rev. 25 (1), 38–47. 10.1016/j.fbr.2011.01.001

[B13] Jiang Y. Tong G. Yin H. Xiong N. (2019). A pedestrian detection method based on genetic algorithm for optimize XGBoost training parameters. IEEE Access 7, 118310–118321. 10.1109/access.2019.2936454

[B14] Kamath U. De Jong K. Shehu A. (2014). Effective automated feature construction and selection for classification of biological sequences. PloS one 9 (7), e99982. 10.1371/journal.pone.0099982 25033270 PMC4102475

[B15] Klein A. Falkner S. Bartels S. Hennig P. Hutter F. (2017). “Fast bayesian optimization of machine learning hyperparameters on large datasets,” in Artificial intelligence and statistics (PMLR), 528–536.

[B16] Kõljalg U. Nilsson R. H. Abarenkov K. Tedersoo L. Taylor A. F. Bahram M. (2013). Towards a unified paradigm for sequence‐based identification of fungi. Mol. Ecol. 22, 5271–5277. 10.1111/mec.12481 24112409

[B32] Lu Y.-Y. Ao Z.-H. Lu Z.-M. Xu H.-Y. Zhang X.-M. Dou W.-F. (2008). Analgesic and anti-inflammatory effects of the dry matter of culture broth of *Termitomyces albuminosus* and its extracts. J. Ethnopharmacol. 120 (3), 432–436. 10.1016/j.jep.2008.09.021 18948177

[B17] Meharunnisa M. Sornam M. (2022). “CatBoost encoded tree-based model for the identification of microbes at genes level in 16S rRNA sequence,” in Communication and intelligent systems: Proceedings of ICCIS 2021 (Singapore: Springer Nature Singapore), 1137–1156.

[B18] Meher P. K. Sahu T. K. Gahoi S. Tomar R. Rao A. R. (2019). funbarRF: DNA barcode-based fungal species prediction using multiclass Random Forest supervised learning model. BMC Genet. 20 (1), 2–13. 10.1186/s12863-018-0710-z 30616524 PMC6323839

[B19] Mossebo D. C. Njounkou A. L. Piatek M. Kengni B. Diasbe M. D. (2009). *Termitomyces striatus* f. pileatus f. nov. and f. brunneus f. nov. from Cameroon with a key to central African species. Mycotaxon 107 (1), 315–329. 10.5248/407.315

[B20] Pedregosa F. Varoquaux G. Gramfort A. Michel V. Thirion B. Grisel O. (2011). Scikit-learn: machine learning in Python. J. Mach. Learn. Res. 12, 2825–2830.

[B21] Pegler D. N. Vanhaecke M. (1994). Termitomyces of southeast asia. Kew Bull. 49, 717–736. 10.2307/4118066

[B22] Prokhorenkova L. Gusev G. Vorobev A. Dorogush A. V. Gulin A. (2018). “CatBoost: unbiased boosting with categorical features,” in NIPS'18: Proceedings of the 32nd International Conference on Neural Information Processing Systems.

[B23] Ren X. Guo H. Li S. Wang S. Li J. (2017). “A novel image classification method with CNN-XGBoost model,” in Digital Forensics and Watermarking: 16th International Workshop, IWDW 2017, Magdeburg, Germany, August 23-25, 2017 (Springer International Publishing), 378–390. Proceedings 16.

[B24] Robson P. B. (2022). MathFeature: feature extraction package for DNA, RNA and protein sequences based on mathematical descriptors. Briefings Bioinforma. 23, 1–10. 10.1093/bib/bbab434 PMC8769707 34750626

[B25] Roe A. D. Rice A. V. Bromilow S. E. Cooke J. E. K. Sperling F. A. H. (2010). Multilocus species identification and fungal DNA barcoding: insights from blue stain fungal symbionts of the mountain pine beetle. Mol. Ecol. Resour. 10, 946–959. 10.1111/j.1755-0998.2010.02844.x 21565104

[B26] Schloss P. D. Westcott S. L. Ryabin T. Hall J. R. Hartmann M. Hollister E. B. (2009). Introducing mothur: open-source, platform-independent, community-supported software for describing and comparing microbial communities. Appl. Environ. Microbiol. 75 (23), 7537–7541. 10.1128/AEM.01541-09 19801464 PMC2786419

[B27] Schoch C. L. Seifert K. A. Huhndorf S. Robert V. Spouge J. L. Levesque C. A. (2012). Nuclear ribosomal internal transcribed spacer (ITS) region as a universal DNA barcode marker for Fungi. Proc. Natl. Acad. Sci. 109 (16), 6241–6246. 10.1073/pnas.1117018109 22454494 PMC3341068

[B28] Somervuo P. Koskela S. Pennanen J. Henrik Nilsson R. Ovaskainen O. (2016). Unbiased probabilistic taxonomic classification for DNA barcoding. Bioinformatics 32 (19), 2920–2927. 10.1093/bioinformatics/btw346 27296980

[B29] Van Velzen R. Weitschek E. Felici G. Bakker F. T. (2012). DNA barcoding of recently diverged species: relative performance of matching methods. PloS one 7 (1), e30490. 10.1371/journal.pone.0030490 22272356 PMC3260286

[B30] Venkatachalapathi A. Paulsamy S. (2016). Exploration of wild medicinal mushroom species in walayar valley, the southern western ghats of coimbatore district Tamil nadu. Mycosphere 7 (2), 118–130. 10.5943/mycosphere/7/2/3

[B31] White T. J. Bruns T. Lee S. J. W. T. Taylor J. (1990). Amplification and direct sequencing of fungal ribosomal RNA genes for phylogenetics. PCR Protoc. a guide methods Appl. 18 (1), 315–322.

